# Associations between 3D surface scanner derived anthropometric measurements and body composition in a cross-sectional study

**DOI:** 10.1038/s41430-023-01309-4

**Published:** 2023-07-21

**Authors:** Manuel Guarnieri Lopez, Katarina L Matthes, Cynthia Sob, Nicole Bender, Kaspar Staub

**Affiliations:** 1 Institute of Evolutionary Medicine, University of Zurich, Zurich, Switzerland; 2 Institute for Environmental Decisions, Consumer Behavior, ETH Zurich, Zurich, Switzerland

**Keywords:** Epidemiology, Epidemiology

## Abstract

**Background:**

3D laser-based photonic scanners are increasingly used in health studies to estimate body composition. However, too little is known about whether various 3D body scan measures estimate body composition better than single standard anthropometric measures, and which body scans best estimate it. Furthermore, little is known about differences by sex and age.

**Methods:**

105 men and 96 women aged between 18 and 90 years were analysed. Bioelectrical Impedance Analysis was used to estimate whole relative fat mass (RFM), visceral adipose tissue (VAT) and skeletal muscle mass index (SMI). An Anthroscan VITUSbodyscan was used to obtain 3D body scans (e.g. volumes, circumferences, lengths). To reduce the number of possible predictors that could predict RFM, VAT and SMI backward elimination was performed. With these selected predictors linear regression on the respective body compositions was performed and the explained variations were compared with models using standard anthropometric measurements (Body Mass Index (BMI), waist circumference (WC) and waist-to-height-ratio (WHtR)).

**Results:**

Among the models based on standard anthropometric measures, WC performed better than BMI and WHtR in estimating body composition in men and women. The explained variations in models including body scan variables are consistently higher than those from standard anthropometrics models, with an increase in explained variations between 5% (RFM for men) and 10% (SMI for men). Furthermore, the explained variation of body composition was additionally increased when age and lifestyle variables were added. For each of the body composition variables, the number of predictors differed between men and women, but included mostly volumes and circumferences in the central waist/chest/hip area and the thighs.

**Conclusions:**

3D scan models performed better than standard anthropometric measures models to predict body composition. Therefore, it is an advantage for larger health studies to look at body composition more holistically using 3D full body surface scans.

## Introduction

High body fat (especially in the abdomen) is an important risk of diabetes type II, cardiovascular diseases and certain cancers as well as with all-cause mortality [1–3]. Also, it is well known that higher levels of physical activity and consequently higher muscle mass are associated with a lower risk for cardiovascular disease and reduces the risk of loss of mobility and mortality, particularly in the elderly [4–6].

The most precise direct measurements of body compositions are usually made by imaging techniques, such as dual-energy X-ray absorptiometry (DEXA), magnetic resonance imaging (MRI) or computer tomography (CT), which can scan the body internally [7, 8]. However, these techniques are time- and cost-consuming and (may) expose the body to invasive X-ray radiation. Due to the ease of handling, the high measurement speed and the transportability of the measuring device, bioelectrical impedance analysis (BIA) is a validated and proven alternative to invasive methods in some study settings [9–11]. Also, the BIA technique has improved over the last decades and new devices are reaching good accuracy levels as compared to the standard imaging methods [9–11].

Most epidemiological studies use standard anthropometry to assess body shape and estimate the Body Mass Index (BMI, kg/m^2^) of the participants However, BMI is a simple and suboptimal indicator of individual body fatness [12, 13], as it is unable to distinguish between weight linked to fat mass and weight linked to lean mass [7, 14, 15]. Thus, BMI does not allow conclusions about fat distribution, which in turn is crucial for the assessment of individual health risk [16, 17]. Other anthropometric body shape measurements such as waist circumference (WC), waist-to-hip (WHR), and waist-to-height (WHtR) ratios are used as proxies for central abdominal fat [18–20]. However, even when trained and qualified personnel perform these measurements and follow standard operating procedures (on posture, breathing position, tape positioning and tension [10, 19, 21]), the acquisition of waist and hip circumference is a time-consuming process that can be subjected to considerable intra- and inter-individual variation [10, 22, 23].

During the last decade, a new method has surfaced as an attractive digital alternative to anthropometrically assess body size and shape from the outside using three-dimensional (3D) photonic surface scan technology [24–26]. The 3D body scanner is a non-invasive, non-contact, harmless, laser-based system that uses cameras surrounding the body to capture information and calculate a detailed body shape map through optical triangulation. The body surface is scanned in approximately 13 seconds and a 3D image of the body topography is produced. The device is able to automatically determine more than 150 body measurements like numerous circumferences, linear dimensions, or regional volumes. A number of validation studies has shown the applicability of the scan technique in an epidemiological setting by comparing scans with manual measurements (e.g., waist or hip circumferences) [27–30]. Good feasibility, reliability and validity of the scans were shown in these studies, and the correlations with parameters linked to metabolic syndrome were comparable to those of studies using manual measurements [28].

Over the last years, the number of publications validating the body scan technology against body composition only slowly increased. However, as Ng et al. [31] state, further studies are justified to elucidate relationships between body shape and composition across sex, age, BMI groups, ethnicity, etc. In order to use 3D scans to assess fat and lean tissue and thus predict cardio-vascular diseases risk, the technology should be calibrated with body composition first to see which aspects of external body shape (circumferences, surfaces, volumes, total and segmental, etc.) in which combination (ratios, etc.) predict best internal body composition. Previous studies have shown that belly circumference and middle hip circumference are important predictors of body fat content and forearm volume and calf volume are good predictors of skeletal muscle mass [30, 32]. However, these studies were carried out on a homogeneous sample of young men only. It is therefore necessary to verify which 3D scan measurements correlate best with body composition in a more heterogenous study population and to study the possible difference in predictors according to sex and age. Furthermore, it is also important to ensure that the combination of measurements as produced by the 3D scan predicts body composition better than conventional anthropometric measurements.

The aim of the present study was to examine which combination of 3D body scanner measurements, together with various socio-demographic and lifestyle variables, best predicted body composition (fat and muscle mass) as measured by BIA in a cross-sectional sample of 96 women and 105 men aged between 18 and 90 years stratified by sex. The second aim was to examine if a combination of 3D scanner parameters produced better predictions of fat and muscle mass than conventional single anthropometric measures.

## Methods

In this article, we use data from a study completed in 2019, which has previously been analysed in a different context [33]. This data set consists of 242 participants which were enrolled from an ongoing national nutrition study (Swiss Food Panel 2.0) through written invitations, mailing lists from scientific communication events, and media announcements to the general population in the Zurich area, Switzerland. To be included in the study, participants had to be at least 18 years old and had to have a good understanding of the German language. Prior to data collection, the study procedures were explained in a written and oral way to the participants and informed consent was obtained. The study was approved by the Ethics Committee of ETH Zurich (EK 2019-N-08).

### Examination battery

The examinations included several steps: First, written self-reported questionnaires developed for previous studies were used to obtain self-reported information on socio-demographic and lifestyle factors, as well as questions on food frequency [34, 35]. Second, an 8-point bioelectrical impedance analysis (BIA) (Seca mBCA 515, Seca AG, Reinach, Switzerland) was utilized to evaluate the total body fat, visceral fat as well as skeletal muscle mass of the participants. The Seca mBCA 515 device has been verified in various studies [36–38] and successfully used in other publications that compare 3D body scans with BIA [28, 39]. Participants stood on the four foot-electrodes barefoot and put both hands on the four hand electrodes. Third, 3D full body surface scans of the participants were performed using a semi-mobile Anthroscan VITUSbodyscan body scanner (Human Solution, Kaiserslautern, Germany) This scanner model is equipped with four eye-safe lasers, eight cameras, and acquires up to 300 data points per cm^2^ as a 3D point cloud, based on optical triangulation. Body scanner derived measurements were acquired using the scanner software (Anthroscan 2016, Version 3.5.3) which automatically calculates 150 standard measurements (ISO 7250 / ISO 8559 and DIN EN ISO 20685) including height, weight and a large number of distances and circumferences and volumes. Following the manufacturer’s instructions, the scanner was calibrated daily before data collection. Participants were briefed and measured according to the standard position (standing up straight, feet positioned on a mark on the scanner platform (ca. 30 cm apart), arms slightly bent at the elbow and held slightly apart from the body, head in accordance to the Frankfurt Horizontal Plane). Volunteers were asked to exhale and not to breathe in during the scan process, which was about 10 seconds. Only form-fitting underwear and a tight-fitting bathing cap were worn during the procedure.

### Dependent variables

For body composition, the BIA output measures visceral fat mass (VAT, kg) and relative fat mass (RFM, % of total body weight) were included in the study. Similar to other studies [40], we calculated the skeletal muscle mass index (SMI) by dividing skeletal muscle mass (SMM, kg) by the square of body height (m).

### Independent variables

On the side of the independent variables (IV), we proceeded as follows: First, we extracted the classical measures for body shape from the 3D scanner data: BMI (kg/m^2^, calculated using height and weight), waist circumference (WC, cm), and waist-to-height-ratio (WHtR, cm/cm). Second, we pre-selected 30 variables from the approximately 150 standard measurements provided by the body scan software that might be relevant for predicting the respective dependent variables (i.e., variables representing various body lengths, girths and volumes). The excluded scan-measurements were mostly textile-specific or redundant measurements. For example, only lengths, circumferences and volumes of the left side limbs were selected. Furthermore, several nearly identical measurements for belly/waist area were excluded (see Supplementary Table 1 for more details).

From the questionnaire, four socio-demographic and lifestyle variables were chosen: age, education category, free-time physical activity and diet. All lifestyle variables were self-reported by the participants. Education was given in following categories 1. mandatory education, 2. basic education, 3. professional training, 4. high school, 5. higher professional studies, 6. higher education, and 7. university. For subgroup size reasons, the data were dichotomised in primary / secondary education [1–4] and tertiary education [5–7]. Physical activity in leisure time was asked as follows: "Please describe your physical activity in leisure time". with the following with the following possible answers: 1. very light, 2. light, 3. moderate, 4. heavy, 5. very heavy. For subgroup size reasons, the data were grouped into three categories in light [1, 2], moderate [3], and heavy [4, 5]. From the food frequency questions, the Diet Quality Index (as described by Hagmann et al. and Sob et al. [41, 42]) was calculated from five food categories: fruits, vegetables, wholegrain products, meat, and sweet/salty snacks. A point was given if the suggested amount for each group was achieved, using the officially suggested minimum or maximum weekly intake as the threshold value. A rating ranging from 0 to 5 was established to indicate the overall healthiness of the diet. [10]. For subgroup size reasons, the score was grouped into three categories in rather unhealthy eating pattern (0–1), medium eating pattern [2, 3], and rather healthy eating pattern [4, 5].

### Statistical analysis

All analyses were performed separately by sex. Spearman’s rank correlation coefficients of independent variables (IV) with RFM, SMI, respectively, were calculated. To reduce the number of possible IV that could predict the selected dependent variables, backward elimination was performed starting with the full model. Backward elimination was preferred to forward selection because of the collinearity between some variables [43]. To check the stability of the selected IV, we repeated the model selection for 2000 bootstrapping samples and calculated the median of bootstrapped regression coefficients. Since the distribution of the bootstrapped regression coefficients was not normal distributed, bias-corrected and accelerated (bca) confidence intervals were calculated [44]. The IV selection was then performed in two steps [45]. First, the bootstrap inclusion frequencies were calculated to quantify how likely an IV was selected. Only IV whose inclusion frequency exceeded 70% were taken. Second, of these selected IVs, only those that did not contain positive and negative values in the bca confidence intervals were selected, i.e., variables that clearly had a negative or positive regression coefficient. These finally selected IV were used to perform linear regression on the respective dependent variables. For model validation bootstrap model validation with 2000 resampling iterations were used. The respective adjusted R-squares were displayed and compared with models that used only BMI, WC, or WHtR to predict the respective dependent variable. Regression scatterplots and corresponding Bland-Altman plots of the best fitted models for all three outcomes were shown. All statistical analyses were performed using R version 4.1.2 [46]. The R package “coxed” [47] was used to calculate the bca confidence intervals and the R package “caret” [48] for model validation The code is available at https://github.com/KaMatthes/Bodyscan_variable_selection.git.

## Results

Of the 242 participants, 201 (83.1%) with complete anthropometric scanner and lifestyle data (see Table 1 and in Supplementary Table 1) were included in the analyses (96 women and 105 men). 37 individuals with missing values in one or several socio-demographic and lifestyle variables as well as 4 imperfect body scans with artefacts were excluded. Male participants were by average 56.4 years old (SD 17.8) and thus significantly older than women (average 47.8 years, SD 19.3, *p* = 0.0014 based on an unpaired two-samples Wilcoxon test). Another significant difference between both sexes can be found for physical activity levels (*p* = 0.0069, based on a chi-square test), with men belonging more frequently to the heavy physical activity category than women (54.3% vs. 35.4%). Moreover, men more frequently belonged to the unhealthy diet category than women (60.0% vs. 38.5%). However, there was no significant difference between the two sexes in terms of education levels (*p* = 0.97). Men were by average 176.0 cm tall (SD 7.1 cm) and thus significantly taller (*p* < 0.001) than women 164.8 cm (SD 6.6 cm). Also, men had a higher average BMI than women (26.1 kg/m^2^ vs. 22.9 kg/m^2^, *p* < 0.001). In terms of the WHO categories for BMI, 38.1% of men were overweight (BMI 25.0–29.9 kg/m^2^), and 16.2% were obese (BMI ≥ 30 kg/m^2^). Women were less likely to be overweight: Only 18.8% were overweight, and 2.1% were obese. To some extent these differences are also reflected in WHtR, but less so in the WC.

**Table 1:** Descriptive statistics of the study group (*N* = 201, 105 men and 96 women).

Men				Women	
*N*	%			*N*	%
105		**Total**		96	
		**Age (years)**			
17	16.2	<36		33	34.4
46	43.8	36–65		41	42.7
42	40	>65		22	22.9
105	100	Total		96	100
		**Education**			
37	35.2	Primary & secondary		35	36.5
68	64.8	Tertiary		61	63.5
105	100	Total		96	100
		**Physical activity**			
16	15.2	Light		12	12.5
32	30.5	Moderate		50	52.1
57	54.3	Heavy		34	35.4
105	100	Total		96	100
		**Diet**			
63	60	Unhealthy		37	38.5
36	34.3	Medium		43	44.8
6	5.7	Healthy		16	16.7
105	100	Total		96	100
		**BMI (kg/m**^**2**^**)**			
0	0	<18.5		4	4.2
48	45.7	18.5–24.9		72	75
40	38.1	25.0–29.9		18	18.8
17	16.2	≥30		2	2.1
105	100	Total		96	100
		**WHtR (cm/cm)**			
40	38.1	≤0.5		57	59.4
43	41	0.51–0.6		32	33.3
22	21	>0.6		7	7.3
105	100	Total		96	100
		**WC (cm)**			
56	53.3	<94.0	<80.0	58	60.4
25	23.8	94.0–101.9	80.0–87.9	18	18.8
24	22.9	≥102.0	≥88.0	20	20.8
		**WHR (cm/cm)**			
54	51.4	<0.90	<0.80	61	63.5
33	31.4	0.90–0.99	0.80–0.84	14	14.6
18	17.1	>1.00	>0.85	21	21.9
105	100	Total		96	100
***N***	**mean (sd)**			***N***	**mean (sd)**
		**Visceral adipose tissue (kg) VAT**			
105	2.57 (1.64)	Total		96	0.88 (0.68)
		**Relative fat mass (%) RFM**			
105	23.31 (6.86)	Total		96	30.51 (6.9)
		**Skeletal muscle mass index (kg/m**^**2**^**) SMI**			
105	9.48 (1.14)	Total		96	7.00 (0.77)

BMI, WHtR, WC and WHR were measured using a 3D scanner and were categorized using the official (WHO-)categories. *N* = absolute frequency, % = relative frequency.

In terms of body composition (Supplementary Table 1), women had by average higher RFM than men (30.5% vs. 23.3%, *p* < 0.001), whereas men had by average significantly higher levels of SMI (9.48 kg/m^2^ vs. 7 kg/m^2^, *p* < 0.001) and visceral adipose tissue (VAT, 2.6 kg vs. 0.9 kg, *p* < 0.001) than women. In all body composition measures there were clear gradients by age groups (Supplementary Fig. 1). Younger men and women had lower fat mass values (RFM and VAT) than older men and women, whereas young people had higher levels of SMI.

Table 2 shows the IV selected by stepwise backwards model for each sex. Mostly, various volumes, measures in the hip and waist area, BMI, and occasionally measures of the arms and thighs were selected, for all three body composition indicators. The total number of IV ranged from 5 (VAT formen) to 12 (RFM for women).

**Table 2:** Independent variables selected in the multivariate scan models using stepwise backward regressions.

	Men		Women	
**VAT**	**Waist girth (cm)**	Volume Belly (l)	**Upper arm girth (cm)**	Waist girth (cm)
	**Hip girth (cm)**	Volume Hip (l)	**Thigh girth horizontal (cm)**	WHR
	**WHR (cm/cm)**		**Volume Hip (l)**	Bust chest girth horizontal (cm)
			Hip girth (cm)	Neck to waist center back (cm)
**RFM**	**Volume Belly (l)**	WHR (cm/cm)	**Volume Hip (l)**	Volume Belly (l)
	**Volume Thigh (l)**	Mid neck girth (cm)	**Waist girth (cm)**	Hip girth (cm)
	**Volume Hip (l)**	Volume Chest (l)	**WHtR (cm/cm)**	BMI (kg/m^2^)
	Maximum belly circumference (cm)	High waist girth (cm)	Bust chest girth horizontal (cm)	Upper arm girth (cm)
	Body height (cm)	Forearm girth (cm)	High waist girth (cm)	Mid neck girth (cm)
**SMI**	**Volume Chest (l)**	Volume Belly (l)	**Waist girth (cm)**	Volume Hip (l)
	**Volume Hip (l)**	BMI (kg/m^2^)	**WHtR (cm/cm)**	Mid neck girth (cm)
	**Volume Thigh (l)**	Forearm girth (cm)	**BMI (kg/m**^**2**^**)**	Cross should over neck (cm)
	Thigh girth horizontal (cm)	Maximum belly circumference (cm)		

Bold variable = The three most important variables in each model.

*VAT* visceral adipose tissue, *RFM* relative fat mass, *SMI* skeletal muscle mass index.

When predicting body composition from standard anthropometric measures (BMI, WC, WHtR) using bivariate linear regression models, WC performed best for VAT and RFM in men and women (Table 3). Adding age in a first step and then also lifestyle variables in a second step increased the explained variation of body composition measures. In the standard anthropometry models, the maximum explained variation of VAT is higher for men than for women with respectively *r*^*2*^ = 0.85 and *r*^*2*^ = 0.79. However, the RFM is better explained for women than for men (maximum explained variation *r*^*2*^ = 0.79 vs *r*^*2*^ = 0.69). For SMI and in both sexes, forearm and thigh circumferences performed better as predictors than the standard anthropometric measures, before adding age and lifestyle variable. After adding age and lifestyle variables, BMI performed better in predicting SMI than all the other single anthropometric predictors, achieving the highest explained variation with *r*^*2*^ = 0.76 in men and *r*^*2*^ = 0.63 in women.

**Table 3:** Comparison of univariable and multivariable regression models for the prediction of body composition (as determined by BIA).

		Visceral adipose tissue (kg) VAT					
		Men			Women		
		*N*	r2	RMSE	*N*	r2	RMSE
**Age**	Age	1	0.22	1.48	1	0.52	0.48
**Lifestyle**	Lifestyle	3	0.27	1.46	3	0.10	0.67
**BMI**	BMI	1	0.59	1.06	1	0.43	0.53
	BMI + age	2	0.74	0.84	2	0.68	0.40
	BMI + age + lifestyle	5	0.75	0.83	5	0.67	0.40
**WC**	WC	1	0.84	0.67	1	0.74	0.63
	WC + age	2	0.85	0.67	2	0.79	0.33
	WC + age + lifestyle	5	0.84	0.67	5	0.77	0.35
**WHtR**	WHtR	1	0.77	0.80	1	0.70	0.39
	WHtR + age	2	0.77	0.81	2	0.73	0.37
	WHtR + age + lifestyle	5	0.77	0.81	5	0.70	0.39
**Scan variables**	Scan variable	5	0.86	0.62	8	0.76	0.35
	Scan variables + age	6	0.89	0.55	9	0.81	0.32
	Scan variables + age + lifestyle	9	0.88	0.57	12	0.79	0.33
		**Relative fat mass (%) RFM**					
**Age**	Age	1	0.21	6.28	1	0.47	5.12
**Lifestyle**	Lifestyle	3	0.24	6.14	3	0.11	6.79
**BMI**	BMI	1	0.46	5.13	1	0.55	4.72
	BMI + age	2	0.61	4.35	2	0.75	3.48
	BMI + age + lifestyle	5	0.62	4.31	5	0.76	3.44
**WC**	WC	1	0.69	3.95	1	0.75	3.48
	WC + age	2	0.69	3.90	2	0.79	3.20
	WC + age + lifestyle	5	0.69	3.92	5	0.77	3.34
**WHtR**	WHtR	1	0.63	4.27	1	0.75	3.48
	WHtR + age	2	0.63	4.26	2	0.77	3.39
	WHtR + age + lifestyle	5	0.63	4.27	5	0.75	3.50
**Scan variables**	Scan variable	10	0.75	3.53	12	0.81	3.07
	Scan variables + age	11	0.76	3.41	13	0.82	2.94
	Scan variables + age + lifestyle	14	0.76	3.50	16	0.81	3.10
		**Skeletal muscle mass index (kg/m**^**2**^**) SMI**					
**Age**	Age	1	0.23	1.03	1	0.20	0.71
**Lifestyle**	Lifestyle	3	0.05	1.18	3	0.07	0.78
**BMI**	BMI	1	0.51	0.81	1	0.23	0.70
	BMI + age	2	0.76	0.56	2	0.59	0.51
	BMI + age + lifestyle	5	0.77	0.55	5	0.63	0.48
**WC**	WC	1	0.14	1.10	1	0.02	0.78
	WC + age	2	0.54	0.79	2	0.38	0.63
	WC + age + lifestyle	5	0.54	0.79	5	0.43	0.61
**WHtR**	WHtR	1	0.12	1.10	1	0.02	0.79
	WHtR + age	2	0.56	0.77	2	0.38	0.62
	WHtR + age + lifestyle	5	0.57	0.76	5	0.43	0.60
**Girth forearm**	Girth forearm	1	0.64	0.70	1	0.28	0.67
	Girth forearm + age	2	0.74	0.59	2	0.48	0.57
	Girth forearm + age + lifestyle	5	0.73	0.60	5	0.50	0.56
**Girth Thigh**	Girth Thigh	1	0.55	0.78	1	0.38	0.62
	Girth Thigh + age	2	0.55	0.78	2	0.43	0.60
	Girth Thigh + age + lifestyle	5	0.52	0.81	5	0.45	0.59
**Scan variables**	Scan variable	8	0.83	0.48	6	0.60	0.50
	Scan variables + age	9	0.85	0.44	7	0.64	0.48
	Scan variables + age + lifestyle	12	0.85	0.44	10	0.64	0.48

The results are shown for the validated boot strapping models. In the scan variable models, the relevant predictors were selected from 30 scanned standard measurements using stepwise backward model. The fit of each model is given as explained variation (adjusted r2). “Lifestyle” includes 3 variables:education level, physical activity level and diet. “*N*” indicates the number of variables included in the model.

*N* numbers of variables included in the model, *r2* explained variation, *RMSE* root mean square error.

**Fig. 1:**
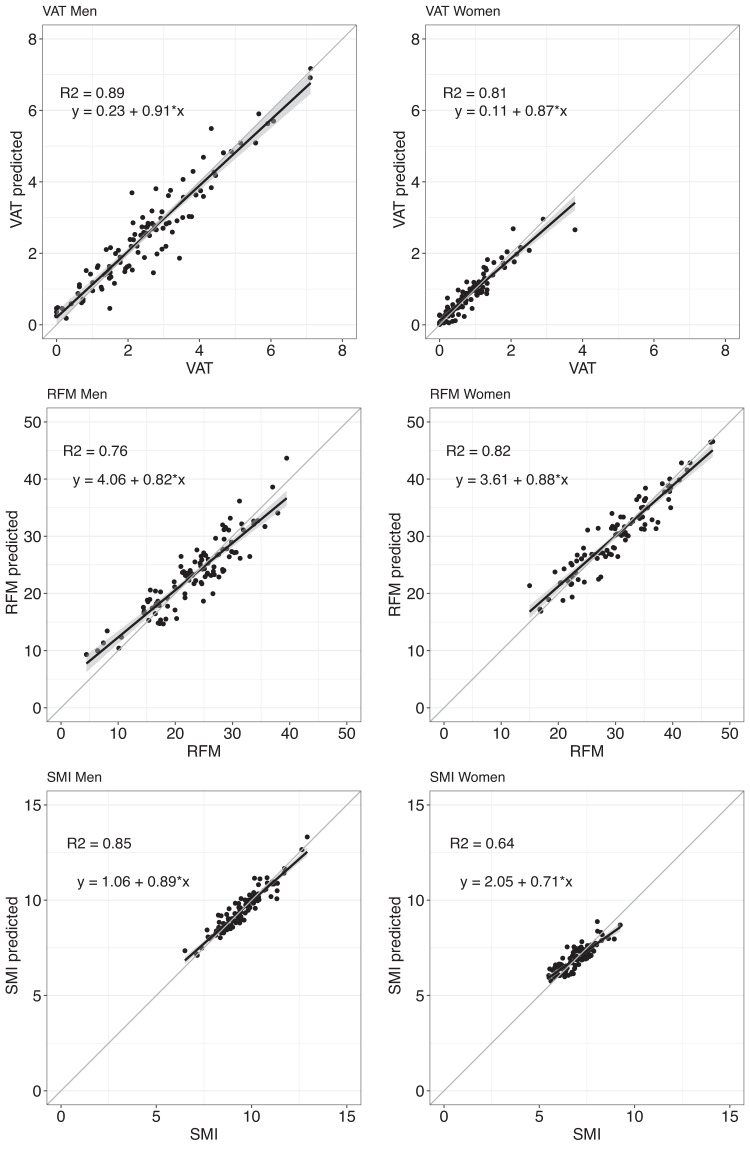
Scatterplot comparisons between the measured body composition values and the predicted values of the best fitted model. VAT visceral adipose tissue (VAT, kg), RFM relative fat mass, SMI skeletal muscle mass index.

**Fig. 2:**
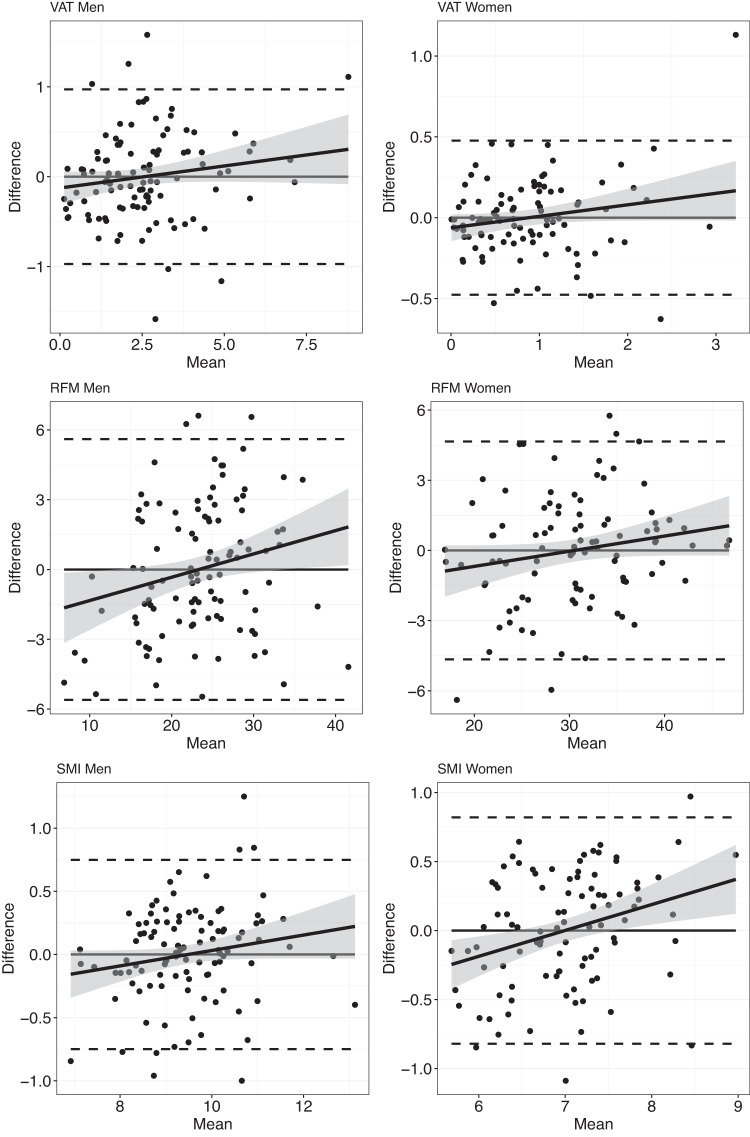
Bland-Altman plots of the measured body composition values and the predicted values of the best fitted model. VAT visceral adipose tissue (VAT, kg), RFM relative fat mass, SMI skeletal muscle mass index. The horizontal line indicates the mean difference between measured and predicted values, the dashed horizontal lines indicate the 95% limits of agreement (Mean + 1.96*SD, Mean – 1.96*SD).

When using the all selected IV (displayed in Table 2) the explained variation for men increased between 4% (VAT) and 10% (RFM) compared to the explained variations from the standard anthropometrics models (after adding age and lifestyle variable). For women an increase between 2% (VAT) and 14% (SMI) was observed. Moreover, RMSE from multivariable scanner-based models decreased in comparison to those from standard anthropometrics models. Figures 1, 2 show the regression scatterplots and the Bland-Altman plots, respectively, of the best fitted models of Table 3. The scatterplots (Fig. 1) indicate linear relationships between the measured and predicted values. However, a proportional bias was observed in the Bland-Altman plots for all values (Fig. 2), but still within the confidence intervals and only a few outliers.

## Discussion

The aim of this study was to assess whether a combination of various measures from 3D body scans in a heterogenous sample of 201 males and females had better predictive power of body composition than traditional anthropometric measures such as BMI, WC and WHtR. We demonstrate that specific combinations of 3D scan measures performed better and that the explained variation was consistently higher than that of traditional anthropometric measures. We also showed that the addition of age as well as socio-demographic variables systematically increased the explained variation in body composition. Our results also highlight that these aspects are sex dependent, and that the selected scanner measures can vary. Overall, we show that there is an advantage to including more aspects of body shape beyond classic anthropometric measures. Here, 3D surface scans certainly bring an advantage in time and precision.

With our study, we contribute to a handful of already published studies that show that adding 3D scanner measurements increase predictive power of body composition. For example, in a study of 1204 volunteers, an index obtained from various 3D scan measures had a better correlation with metabolic risk factors than BMI and WHR alone [49]. A study to determine predictive equations for body fat composition found that, traditional methods and 3D scan methods performed equally for the prediction of total and subcutaneous adiposity. However, for visceral adiposity, 3D scan measures provided a better prediction model [50]. Another study involving 456 healthy adults showed that principal components (PCAs) from 3D scans can predict body composition with greater accuracy than traditional anthropometric models [51]. Another study have compared 4 commercially available 3D scanners to predict body composition and have shown that all scanners reliable estimate body composition [52]. In various studies using data from children and adults of the Shape up! Study in Hawaii, Wong et al. and Bennett et al. have shown that 3D scan methods predicts body composition with high precision [53–56]. A previous study on a homogeneous group of young Swiss men showed that multivariable regression models including 3D scans for the prediction of body composition had a better predictive value than univariable models based on classical anthropometric measurements [30]. However, as in our study, it could be shown that WC and WtHR are also good predictors for estimating VAT and RFM, whereas skeletal muscle mass could not be estimated so well with WC and WtHR, showing a clear advantage of the 3D scanner measurement.

Our study goes beyond these previous studies by also looking at aspects of muscle mass in a heterogeneous sample. To relativize skeletal muscle mass, we followed other studies [40] and used SMI. This index showed a high correlation with grip strength, cardiopulmonary endurance, leg endurance, gait speed, and flexibility.

Our results suggest that selected scanner measures for predicting body composition are sex-specific. This is consistent with the fact that for a given BMI, men have more lean mass and women generally have a larger proportion of body mass from fat [57]. Moreover women are more likely to deposit fat subcutaneously and on their lower extremities while men have more visceral and hepatic adipose tissue in the abdomen [58]. A study aimed at developing prediction equations for the measurement of total abdominal, subcutaneous and visceral adiposity by 3D scanning showed that sex was always included as a variable in the predictions [50]. In addition, another study creating mathematical equations for the prediction of total and regional (trunk, legs) body fat concluded that sex was one of the most critical components that was incorporated into most equations [59]. These results imply that sex is an important co-factor in predicting adiposity, as there are differences in the distribution and accumulation of fat between men and women. In a cross-sectional study of 9617 adults which aimed were to investigate the relation of body shape and BMI and to examine associations between age, sex, and shape, BMI was significantly associated with chest and waist dimensions in men and with hips and bust dimensions in women [60]. Overall, these results suggest that because of the fundamental biological differences between men and women in body composition, the two sexes should be analysed separately in anthropometric studies.

By adding age as an independent variable in our models, the explained variation of the different body composition aspects systematically increased. This is consistent with the fact that body composition changes over the life course, when usually a decrease in fat-free mass and an increase in percent body fat with aging is found in many populations [61]. Two studies looking at the correlation between three-dimensional scanner anthropometric measures with metabolic risk factors confirmed the important role of age as a co-factor [49, 62]. However, three studies with a narrower age span than ours also showed that age was not a significant contributor in these samples of men and women younger than 65 years of age [50, 59, 63]. In addition, a cross-sectional study has shown that associations of body shape with age were significantly stronger in women than in men [60]. This is also the case in our study, when the prediction improved more in women than in men when adding age to the models.

The software, which together with the scanner device allows the processing of the scans (in our case this was Anthroscan VITUSbodyscan), usually produces about 150 standard measures via standard algorithms. But these many standard measurements include some redundant measurements (e.g., length of the right and left leg) as well as some measurements that are especially valuable for the clothing industry and are less relevant in a health-related context (e.g., the length of the arm until the back of the neck). Also, many of these measures are correlated when used as independent explanatory variables [30]. In the existing similar comparative studies [30, 31, 59], the different research teams have dealt with this initial situation differently and have used different methods to pre-select variables and predict body composition using measurements derived from 3D scans. All in all, in our present study, we found similar strength of association between scanner measurements and body composition aspects as in comparable studies (the best models also achieved *r*^*2*^ between 0.60 and 0.95 in the literature). Regarding methods, other teams took slightly different although similar paths, when usually stepwise regressions (using different criteria) [30, 31, 59], principal component analysis (PCA) [51], or data-driven machine learning approaches were used [64, 65]. The selected measures to predict body fat varied more between studies. However, in most cases, volume and circumferences in the central waist/chest/hip area and the thighs were selected [30, 31, 59], which led to the strongest associations with body fat.

Our study has some limitations. We used a BIA device to assess body composition, which is not the gold standard for measuring body composition in clinical settings [66–69]. One of the limitations of the technique is that the calculation of body composition depends on population-specific equations and that accuracy of measurements is not absolutely precise [70]. Furthermore, devices from different manufacturers provide different results [71]. This makes it difficult to compare results from different manufacturers. However, the latest generation BIA devices (like the Seca mBCA 515 device we used) show very good results in validation studies, although some discrepancies may occur especially with visceral adipose tissue fat [36]. Another limitation for the 3D Scanner is, that we could not correct the results for the residual lung volume. Furthermore, to our knowledge, there are no validation studies of the body volumes determined by the scanner yet, so that we cannot estimate their influence. In our non-clinical field work context, it was not possible to use invasive and time-consuming methods such as DEXA. Moreover, we conducted the study on a relatively small number of subjects. It is important to note that the age distribution of the subjects is not homogeneous either between age groups or between the sexes. Only 16.2% of the men were younger than 36 years and 40% were older than 65 years, while 34.4% of the women were younger than 36 years and only 22.9% were older than 65 years. With a larger number of participants and better homogeneity between age groups, it would be possible to study the relationship between body composition and 3D body scan measurements by stratifying by sex and age group.

## Conclusions

We show that there is an advantage to including more aspects of body shape beyond classic anthropometric measures to estimate body composition. 3D surface scans are certainly one possible way to achieve this with high precision in a minimal time. However, given the fundamental biological differences between the sexes, different scan measurements should be used between men and women to obtain a more accurate assessment of body composition. In addition, the best predictors of body composition also vary with age, mirroring changes in the distribution of fat and muscle mass over the life course. Therefore, further studies with more participants and a broader and homogeneous age distribution are needed in the future.

Also, in terms of future studies in this sub-field, various studies use different statistical methods to assess which preselection of scanner measurements in which combination predict body composition. The statistical measure used to describe the strength of association also varied from study to study. Thus, the various studies, although using the same or very similar scanning equipment, are currently difficult to compare directly without access to original data. The groups involved should develop a standard reporting protocol or share original data so that meta-studies and more general statements can be made across individual studies.

### Supplementary information

Supplement

## Data Availability

The data and code underlying this manuscript are publicly available via zenodo and GitHub: 10.5281/zenodo.7108758. https://github.com/KaMatthes/Bodyscan_variable_selection.git.
