# Supplementary material for: Associations between 3D surface scanner derived anthropometric measurements and body composition in a cross-sectional study
Source: Eur J Clin Nutr. 2023 Jul 21;77(10):972–81. doi: 10.1038/s41430-023-01309-4 (PMC10564621; doi:10.1038/s41430-023-01309-4)

Supplement

Supplement Table 1: Descriptive statistics of all included continuous variables. Sd=Standard deviation; Rho=Spearman rank correlation Rho with RFM and SMI, only Rho>-/+0.5 are reported, bold numbers=Rho>=+/-0.80.

	Men						Women					
	Mean	sd	Min.	Max.	Rho RFM	Rho SMI	Mean	sd	Min.	Max.	Rho RFM	Rho SMI
Relative fat mass value (RFM, %)	23.32	6.85	4.45	39.47	**1**		30.51	6.91	14.97	47	**1**	
Absolute fat mass value (AFM, kg)	19.26	7.62	3.29	46.43	**0.94**		19.24	6.33	7.34	43.97	**0.93**	
Skeletal muscle mass index (SMI, kg/m2)	9.48	1.14	6.5	12.92		**1**	7	0.77	5.5	9.25		**1**
Skeletal muscle mass value (SMM, kg)	29.41	4.12	20.19	41.18		0.78	19.04	2.7	13.48	26.07		**0.82**
Visceral adipose tissue value (VAT, kg)	2.57	1.64	0	9.33	**0.88**		0.88	0.68	0	3.79	**0.9**	
Age (years)	56.4	17.79	20	90			47.76	19.31	18	86	0.7	
Body height (cm)	176.01	7.07	161	197.2			164.76	6.57	150.8	183.5		
Weight (kg)	80.78	11.22	60.6	117.7	0.55	0.65	62.09	8.35	45.1	93.6		0.55
BMI scan (kg/m2)	26.11	3.61	20.15	39.75	0.67	0.71	22.89	2.9	18.11	32.08	0.69	
Waist girth (cm)	93.68	11.26	74.1	126.8	**0.82**		80.02	10.37	62.4	111.6	**0.86**	
WHtR	0.53	0.07	0.4	0.74	**0.81**		0.49	0.07	0.37	0.67	**0.86**	
WHR	0.9	0.09	0.72	1.1	0.71		0.78	0.08	0.65	0.98	0.76	
Distance waist knee (cm)	62.21	2.89	56.3	70.7			57.98	3.25	51	67.2	-0.51	
Hip height (cm)	86.91	5.53	73.2	102.8			79.63	4.97	70.1	91.5		
Breast height (cm)	126.72	5.93	114.2	144.7			116.75	6.15	100.6	133		
Mid neck girth (cm)	39.85	2.99	33	50	0.7		32.79	2.13	29.1	40.2	0.6	
Cross shoulder over neck (cm)	43.64	2.59	37.1	50.1	0.52		40	2.16	35.4	46.3		
Bust chest girth horizontal (cm)	104.42	8.27	88.8	126.8	0.6	0.58	92.54	7.38	80.7	118.7	0.55	
Neck to waist center back (cm)	43.49	1.94	39.6	49.3			39.85	2.11	34.6	45.8		
High waist girth (cm)	92.65	10.76	74.4	122.9	**0.8**		77.74	9.66	62.4	106	0.79	
Hip girth (cm)	103.77	6.48	91.3	132.6		0.6	102.54	6.19	91.2	126.2	0.53	
Maximum belly circumference (cm)	97.24	10.36	78.6	131.5	**0.82**		87.6	8.67	71.3	115.2	**0.8**	
Arm length left (cm)	60.85	3.3	52.8	68			55.04	3.27	48.3	62.2		
Upper arm girth left (cm)	29.88	2.55	23.9	38.7		0.72	27.44	2.19	23.1	33.7	0.54	
Elbow girth left (cm)	27.32	1.71	23.8	32		0.71	24.18	1.54	21.1	28.5	0.59	
Forearm girth left (cm)	27.14	1.86	22.9	32.7		**0.83**	23.36	1.46	20.4	26.8		
Thigh girth left horizontal (cm)	55.65	4.32	45.4	70.2		0.68	55.39	3.69	47.6	66.9		0.6
Knee girth left (cm)	38.98	2.35	34.3	46.2	0.5		37.27	2.27	31	45.1		
calf girth left (cm)	37.91	2.57	30.1	44.8		0.62	35.98	2.16	31.8	43		
min leg girth left (cm)	22.47	1.41	18.6	26.9		0.54	21.23	1.47	18.3	25.5		
Volume Upperarm left (l)	1.69	0.28	1.15	2.77		0.65	1.36	0.22	0.93	1.96		
Volume Forearm left (l)	1.05	0.16	0.71	1.56		0.71	0.71	0.11	0.49	1.03		0.56
Volume Thigh left (l)	6.99	1.14	4.84	11.28		0.52	6.76	1.06	4.79	10.85		
Volume lower leg left (l)	3.14	0.5	2.11	4.92		0.55	2.75	0.42	1.95	4.34		
Volume Chest (l)	23.76	3.35	16.86	32.19	0.66		17.47	3.07	12.08	28.45	0.66	
Volume Belly (l)	7.9	1.99	3.68	14.41	0.53		4.73	1.23	2.26	9.48		
Volume Hip (l)	16.07	3.97	9.83	35.18	0.69		12.66	3	7.02	25.82	0.72	

Supplement Table 2: β Coefficients and 95% bca CI of the finally selected independent variables

**VAT Men**	**β**	**95% bca CI**
Waist girth (cm)	0.59	0.27 - 1.12
Hip girth (cm)	-0.3	-0.56 - 0
WHR (cm/cm)	-38.57	-68.33 - -9.25
Volume Belly (l)	0.16	0 - 0.31
Volume Hip (l)	0.14	0 - 0.34

**VAT Women**	**β**	**95% bca CI**
Bust chest girth horizontal (cm)	0.04	0 - 0.08
Neck to waist center back (cm)	0.07	0 - 0.15
Waist girth (cm)	0.23	0 - 0.49
Hip girth (cm)	-0.08	-0.26 - 0
Upper arm girth (cm)	0.1	0 - 0.19
Thigh girth horizontal (cm)	-0.09	-0.16 - 0
WHR	-11.24	-31.27 - 0
Volume Hip (l)	0.16	0 - 0.3

**RFM Men**		
Body height (cm)	-0.8	-2.12 - 0
Mid neck girth (cm)	0.47	0 - 0.93
High waist girth (cm)	-0.64	-1.43 - 0
Maximum belly circumference (cm)	0.38	0 - 0.82
Forearm girth (cm)	-0.99	-2.03 - 0
WHR (cm/cm)	158.96	0 - 324.7
Volume Thigh (l)	4.29	1.51 - 7.49
Volume Chest (l)	1.3	0 - 2.35
Volume Belly (l)	1.57	0 - 2.64
Volume Hip (l)	1.56	0.59 - 2.78

**RFM Women**		
Mid neck girth (cm)	0.63	0 - 1.18
Bust chest girth horizontal (cm)	0.28	0 - 0.68
Neck to waist center back (cm)	0.92	0 - 1.83
Waist girth (cm)	-4.27	-7.3 - -1.85
High waist girth (cm)	-0.3	-0.73 - 0
Hip girth (cm)	1.63	0 - 2.99
Upper arm girth (cm)	0.59	0 - 1.39
WHtR (cm/cm)	455.26	0 - 1021.29
WHR (cm/cm)	207.22	0 - 369.72
Volume Belly (l)	0.94	0 - 2.24
Volume Hip (l)	1.47	0 - 2.56
BMI (kg/m2)	-3.24	-12.82 - 0

**SMI Men**		
Maximum belly circumference (cm)	-0.06	-0.11 - 0
Forearm girth (cm)	0.14	0 - 0.27
Thigh girth horizontal (cm)	0.1	0 - 0.19
Volume Thigh (l)	-0.67	-1.07 - -0.28
Volume Chest (l)	-0.21	-0.37 - -0.08
Volume Belly (l)	-0.21	-0.36 - -0.07
Volume Hip (l)	-0.22	-0.39 - -0.09
BMI (kg/m2)	0.56	0 - 0.96

**SMI Women**		
Mid neck girth (cm)	-0.09	-0.17 - 0
Cross shoulder over neck (cm)	0.08	0 - 0.16
Waist girth (cm)	0.48	0 - 1
WHtR (cm/cm)	-87.18	-165.9 - 0
Volume Hip (l)	-0.18	-0.33 - 0
BMI (kg/m2)	1.24	0.34 - 2.14

Supplement Figure 1: Boxplots of visceral adipose tissue (VAT, kg), relative fat mass (RFM, %) and skeletal muscle mass index (SMI, kg/m2) according to sex and age groups

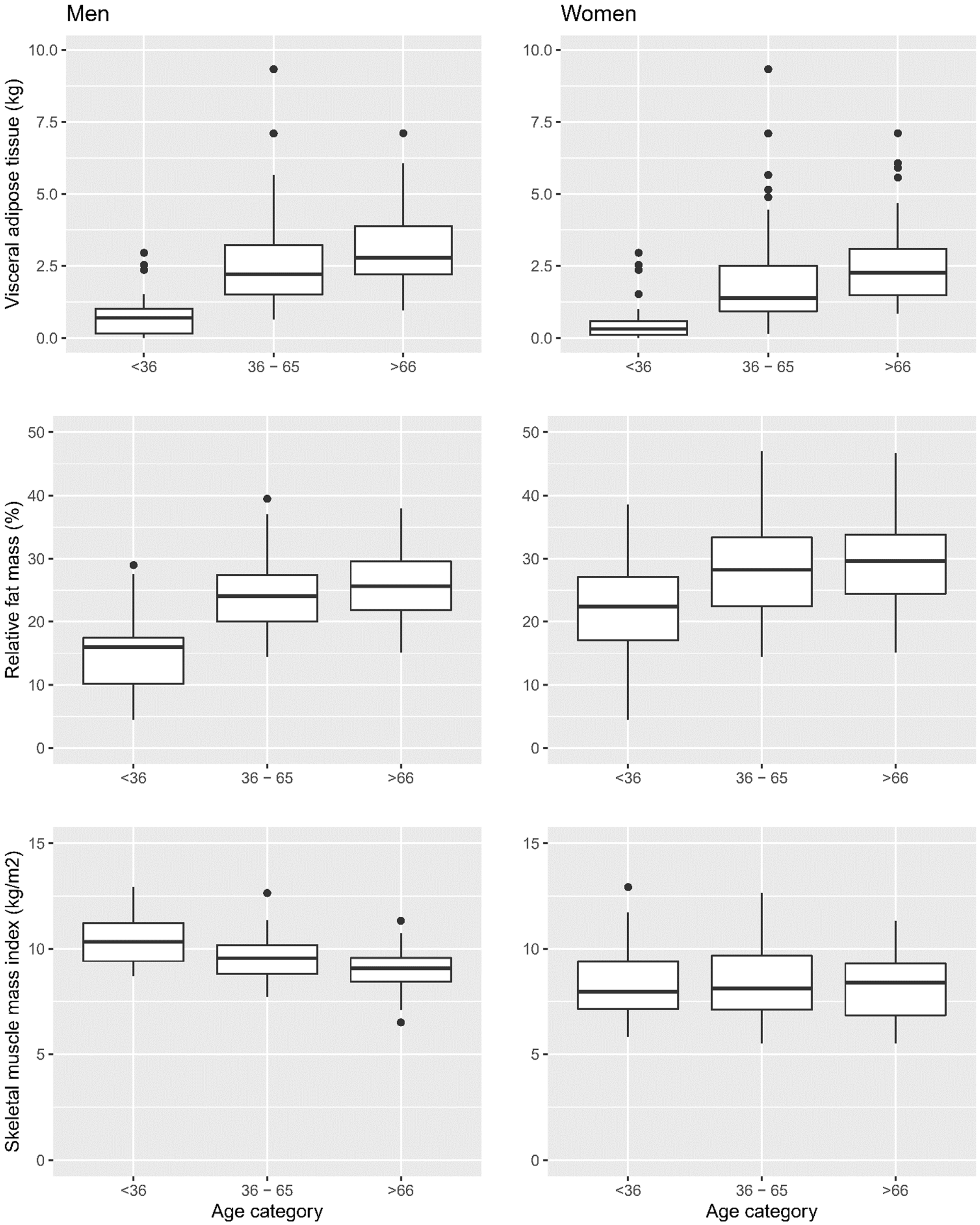

Supplement: Supplementary file 1 — Supplement [file 41430_2023_1309_MOESM1_ESM.pdf]
